# Understanding the Spectrum and Management of Post-Tuberculosis Lung Disease: A Comprehensive Review

**DOI:** 10.7759/cureus.63420

**Published:** 2024-06-28

**Authors:** Sankalp Yadav, Gautam Rawal

**Affiliations:** 1 Medicine, Shri Madan Lal Khurana Chest Clinic, New Delhi, IND; 2 Respiratory Intensive Care, Max Super Speciality Hospital, New Delhi, IND

**Keywords:** anti-tb treatment, tb treatment, mycobacterium tuberculosis (mtb), tb, fev (forced expiratory volume), post tuberculosis lung disease

## Abstract

Post-tuberculosis lung disease (PTLD) poses a significant clinical challenge in regions with a high burden of tuberculosis (TB). This review provides a comprehensive overview of PTLD, encompassing its pathogenesis, clinical manifestations, diagnostic modalities, management strategies, long-term outcomes, and public health implications. PTLD arises from residual lung damage following TB treatment and is characterized by a spectrum of pathological changes, including fibrosis, bronchiectasis, and cavitation. Clinical presentation varies widely, from chronic cough and hemoptysis to recurrent respiratory infections, which are oftentimes a diagnostic dilemma. Radiological imaging, pulmonary function tests, and careful consideration of patient history play pivotal roles in diagnosis. Management strategies involve pharmacological interventions to alleviate symptoms and prevent disease progression, which are influenced by the extent of lung damage, comorbidities, and access to healthcare. Rehabilitation programs and surgical options are available for select cases. Prognosis is influenced by the extent of lung damage, comorbidities, and access to healthcare. Prevention efforts through a TB control program and early detection are crucial in reducing the burden of PTLD. This review stresses the importance of understanding and addressing PTLD to mitigate its impact on individuals and public health systems worldwide.

## Introduction and background

Tuberculosis (TB) remains one of the most significant infectious diseases globally, with a profound impact on public health and socioeconomic development. It is caused by *Mycobacterium tuberculosis *(Mtb). Despite considerable progress in TB control efforts, it continues to pose a formidable challenge, particularly in resource-limited settings [1]. The World Health Organization (WHO) estimates that over 10 million people develop active TB each year, with approximately 1.5 million deaths attributed to the disease annually [2]. Furthermore, the emergence of drug-resistant TB strains further complicates treatment and control efforts [3].

While successful TB treatment can lead to a microbiological cure and clinical resolution of symptoms, it often leaves residual lung damage in its wake [4]. This sequelae, known as post-tuberculosis lung disease (PTLD), encompasses a spectrum of pathological changes in the lungs, including fibrosis, bronchiectasis, and cavitation [5]. PTLD can manifest months to years after the completion of TB treatment and presents a significant clinical challenge due to its variable and often insidious onset.

The prevalence of PTLD is on the rise globally, paralleling the increasing burden of TB [6]. Studies indicate that a substantial proportion of TB survivors develop PTLD, with estimates ranging from 20% to 60% depending on factors such as TB severity, treatment adherence, and host immune response [7]. As TB control efforts improve and more individuals survive the disease, the long-term consequences of PTLD are becoming increasingly evident, stressing the importance of understanding and addressing this condition [8].

The rising prevalence of PTLD has significant implications for healthcare systems worldwide [4]. Not only does it contribute to the overall burden of respiratory diseases but it also imposes substantial costs on healthcare resources, including hospitalizations, medications, and long-term care [7]. Moreover, PTLD can have a profound impact on the quality of life of affected individuals, impairing lung function, reducing exercise capacity, and predisposing them to recurrent respiratory infections [4].

## Review

Pathogenesis of PTLD

PTLD arises from the complex interplay of host immune responses, microbial factors, and tissue remodeling processes following the completion of TB treatment [9,10]. Understanding the pathophysiological changes occurring in the lungs is essential for elucidating the mechanisms underlying PTLD development.

TB treatment aims to eradicate Mtb and minimize lung tissue damage. However, despite successful treatment, residual lung damage often persists, setting the stage for PTLD [5]. One of the important features of PTLD is the formation of pulmonary fibrosis, characterized by excessive deposition of collagen and other extracellular matrix components. Fibrosis develops as a consequence of chronic inflammation and tissue repair processes initiated during active TB infection. Persistent inflammation, coupled with dysregulated wound healing responses, leads to the progressive accumulation of scar tissue, impairing lung function and predisposing to respiratory complications [5,11].

Bronchiectasis, another common feature of PTLD, results from the destruction of bronchial walls and dilation of the bronchi and bronchioles [12]. Cavitation, observed in some cases of TB, contributes to the development of bronchiectasis by causing structural damage to the airways and facilitating the formation of bronchopleural fistulae [5]. These anatomical abnormalities promote mucus retention, chronic bacterial colonization, and recurrent respiratory infections, further exacerbating lung damage in PTLD [13].

The pathogenesis of PTLD is also influenced by host immune responses to Mtb and its antigens. Following TB treatment, the immune system undergoes dynamic changes as it transitions from a state of active infection to a state of quiescence. However, immune dysregulation can occur, leading to persistent inflammation and tissue damage. Altered cytokine profiles, such as elevated levels of tumor necrosis factor-alpha (TNF-α) and transforming growth factor-beta (TGF-β), have been implicated in the pathogenesis of PTLD, promoting fibrogenesis and impairing tissue repair mechanisms [5]. Conversely, regulatory T cells (Tregs) and anti-inflammatory cytokines, such as interleukin-10 (IL-10), play a role in dampening excessive immune responses and promoting tissue repair post-TB [5,14]. However, the balance between pro-inflammatory and anti-inflammatory signals may be disrupted in PTLD, contributing to ongoing lung injury and fibrosis [5]. Overall, the pathogenesis of PTLD is multifactorial, involving a complex interplay of inflammatory, immunological, and tissue remodeling processes.

Causes of PTLD

There are several causes of PTLD. The majority of these are tabulated in Table 1.

**Table 1:** Causes of post-tuberculosis lung disease

Cause	Category	Examples
Pulmonary damage	Fibrosis	A number of individuals treated for tuberculosis develop pulmonary fibrosis.
	Cavitation	Persistent cavities can lead to chronic lung impairment and secondary infections.
	Bronchiectasis	Significant number of individuals treated for tuberculosis develop bronchiectasis.
Infection factors	Persistent *Mycobacterium tuberculosis* infection	Long-term follow-up studies indicate persistent infection in a small percentage of cases.
	Multi-drug-resistant tuberculosis	Multi-drug-resistant tuberculosis rates vary but are a significant concern globally, contributing to treatment failure and increased lung damage.
Treatment complications	Drug-induced lung injury	Isoniazid and rifampicin can cause drug-induced pneumonitis or fibrosis.
	Surgical complications	Lung resections or pleural drainage may lead to long-term respiratory impairment or complications.
Host factors	Immune status	HIV co-infection increases tuberculosis risk and complicates treatment outcomes.
	Coexisting conditions	Chronic obstructive lung disease or diabetes mellitus exacerbate tuberculosis-related lung damage.
Environmental and social factors	Living conditions	Indoor air pollution from biomass fuels increases respiratory infections and delays recovery from tuberculosis.
Individual variability	Access to healthcare	Delays in seeking care contribute to advanced disease presentation and poorer treatment outcomes.
	Genetic predisposition	Genetic variants (e.g., tumour necrosis factor-alpha) may influence susceptibility to severe tuberculosis and subsequent lung damage.
	Age	Older adults are at higher risk for severe tuberculosis and long-term pulmonary sequelae.

Clinical manifestations

PTLD presents with a spectrum of symptoms and signs reflecting the diverse pathological changes occurring in the lungs following TB treatment [4]. Recognizing these clinical manifestations is crucial for the timely diagnosis and management of PTLD.

Common symptoms of PTLD include chronic cough, which may be productive or non-productive and often persists for months to years after completion of TB treatment [15]. Hemoptysis, the coughing up of blood, can occur due to bronchial erosion secondary to bronchiectasis or cavitation [16]. Dyspnea, or difficulty breathing, may develop as lung function deteriorates due to fibrosis and airway obstruction. Recurrent respiratory infections are also common, reflecting impaired mucociliary clearance and a predisposition to bacterial colonization in structurally damaged airways [4].

On physical examination, signs of PTLD may include diminished breath sounds over areas of fibrosis, wheezing due to airway narrowing, and crackles suggestive of airspace disease. Digital clubbing, a manifestation of chronic hypoxia and tissue hypoxia, may be observed in advanced cases.

Advanced radiological imaging, like computed tomography (CT), plays a central role in the evaluation of PTLD. Chest X-rays and CT scans reveal characteristic findings such as fibrotic changes, including linear or reticular opacities, traction bronchiectasis, and honeycombing indicative of advanced fibrosis [5]. Bronchiectasis, manifested by dilated and thickened bronchial walls, may appear as "tram lines" or "signet ring" signs on imaging. Nodular opacities, representing residual granulomas or calcified lymph nodes, may also be observed, particularly in areas of prior TB involvement. However, there are no clinical criteria for chest radiographs or CT scans that would comprehensively define PTLD [5]. Oftentimes, the ambiguity in presentation results in unnecessary treatment reinitiating with anti-TB drugs [4].

The variability in PTLD presentation is influenced by the severity and extent of lung damage, which can vary widely among individuals [9]. While some patients may remain asymptomatic or experience mild respiratory symptoms, others may develop progressive lung dysfunction and respiratory failure. Factors such as the duration and intensity of TB infection, the adequacy of TB treatment, the presence of comorbidities, and the host immune response contribute to this variability [7,9].

Diagnostic approaches

PTLD poses several challenges in diagnosis owing to its diverse clinical manifestations and overlapping features with other lung diseases. Accurate diagnosis requires a comprehensive evaluation incorporating clinical history, radiological imaging, pulmonary function tests, and consideration of past TB treatment.

One of the primary challenges in diagnosing PTLD is its resemblance to other chronic lung diseases, such as chronic obstructive pulmonary disease (COPD), interstitial lung disease (ILD), and bronchiectasis. Similarities in symptoms such as chronic cough, dyspnea, and recurrent respiratory infections can make it difficult to differentiate PTLD from these conditions based solely on clinical presentation. Additionally, radiological findings of fibrotic changes, bronchiectasis, and nodules observed in PTLD may overlap with those seen in other lung diseases, further complicating the diagnosis [4].

Radiological imaging, including chest X-rays and CT scans, plays a central role in the diagnosis of PTLD. Chest X-rays may reveal abnormalities such as fibrotic opacities, cavitation, and bronchiectasis, although they may lack sensitivity for detecting subtle changes. CT scans offer higher resolution and detailed assessment of lung parenchyma, airways, and pleura, enabling the detection of fibrotic changes, bronchiectasis, and nodular opacities characteristic of PTLD. High-resolution CT (HRCT) is particularly valuable in delineating the extent and severity of lung damage in PTLD. Further, positron emission tomography (PET) using 18F-fluorodeoxyglucose (18FDG) as a tracer to show glucose metabolism along with CT imaging constitutes a powerful imaging tool that can give valuable structural information [5,17].

Pulmonary function tests (PFTs) provide objective measurements of lung function and aid in the assessment of disease severity and progression in PTLD [15]. Spirometry, lung volume measurements, and diffusing capacity for carbon monoxide (DLCO) are commonly performed tests that can reveal airflow limitations, restrictive patterns, and impaired gas exchange, respectively [18]. Abnormal PFT results, such as reduced forced expiratory volume in one second (FEV1) and decreased DLCO, may suggest the presence of PTLD and help guide management decisions.

In addition to radiological and pulmonary function assessments, careful consideration of patient history is essential in diagnosing PTLD. A detailed history of prior TB infection, including treatment duration, adherence, and response to therapy, provides valuable clues to the underlying etiology of lung disease. Patients with a history of TB are at increased risk of developing PTLD, particularly if TB treatment is incomplete or inadequately administered [19].

Management strategies

PTLD necessitates a multifaceted approach to management aimed at alleviating symptoms, slowing disease progression, and improving overall quality of life. Management strategies for PTLD encompass pharmacological interventions, rehabilitation programs, and surgical options tailored to individual patient needs and disease severity [4].

Pharmacological Interventions

Pharmacotherapy plays a central role in managing symptoms and preventing disease progression in PTLD. Bronchodilators, including short-acting beta-agonists (SABAs) and long-acting beta-agonists (LABAs), are commonly used to alleviate bronchospasm and improve airflow in patients with concomitant bronchial hyperreactivity. Inhaled corticosteroids (ICS) may be prescribed to reduce airway inflammation and minimize exacerbations, particularly in patients with evidence of eosinophilic inflammation or asthma-like symptoms [4,20].

Mucolytic agents such as N-acetylcysteine (NAC) or hypertonic saline may be utilized to facilitate mucus clearance and reduce sputum production in patients with chronic bronchitis or bronchiectasis [21]. Antibiotics may be indicated for the treatment of acute exacerbations or chronic bacterial colonization, particularly in patients with recurrent respiratory infections.

For individuals with significant dyspnea and impaired gas exchange, supplemental oxygen therapy may be necessary to maintain adequate oxygenation and alleviate hypoxemia. Non-invasive ventilation (NIV) may be considered in selected cases to improve ventilation and relieve respiratory distress.

Rehabilitation Programs

Pulmonary rehabilitation programs play a vital role in optimizing lung function, enhancing exercise capacity, and improving quality of life in patients with PTLD [4]. These programs typically include a combination of exercise training, education, nutritional counseling, and psychosocial support tailored to individual patient needs [22].

Exercise training, including aerobic and resistance exercises, improves cardiorespiratory fitness, muscle strength, and endurance, thereby enhancing functional capacity and reducing dyspnea. Education sessions provide patients with information on disease management, breathing techniques, medication adherence, and self-management strategies to optimize treatment outcomes [22,23].

Nutritional counseling aims to address malnutrition and weight loss commonly observed in patients with advanced PTLD, emphasizing dietary modifications to meet energy requirements, promote weight gain, and optimize nutritional status [4,22]. Psychosocial support helps patients cope with the emotional and psychological challenges associated with chronic lung disease, fostering resilience and enhancing overall well-being.

Surgical Options

In select cases of PTLD with severe complications such as massive hemoptysis, localized lung damage, or refractory respiratory symptoms, surgical intervention may be warranted. Surgical options for PTLD include bronchial artery embolization, lobectomy, segmentectomy, or lung transplantation, depending on the extent and severity of lung involvement and the patient's overall health status [24].

Bronchial artery embolization is a minimally invasive procedure used to control life-threatening hemoptysis by occluding aberrant bronchial arteries that supply bleeding bronchial vessels [25]. Lobectomy or segmentectomy may be considered for localized lung damage or extensive bronchiectasis causing recurrent infections or respiratory compromise [24]. Lung transplantation may be indicated in patients with end-stage PTLD who are refractory to medical and surgical therapies, offering a potential cure for selected individuals.

Overall, the management of PTLD requires a comprehensive approach encompassing pharmacological interventions, pulmonary rehabilitation programs, and surgical options tailored to individual patient needs and disease severity. By addressing symptoms, slowing disease progression, and improving overall quality of life, these management strategies aim to optimize outcomes and enhance well-being in patients affected by PTLD.

Long-term outcomes and prognosis

The prognosis of PTLD is influenced by a myriad of factors, including the extent of lung damage, the presence of comorbidities, and access to healthcare resources. Understanding these factors is crucial to predicting disease progression and optimizing patient outcomes.

Factors Influencing Prognosis

The extent and severity of lung damage play a pivotal role in determining the prognosis of PTLD. Patients with extensive fibrosis, bronchiectasis, and cavitation are at higher risk of respiratory compromise, recurrent infections, and progressive decline in lung function [5]. Comorbidities such as COPD, cardiovascular disease, and diabetes mellitus further contribute to the complexity of PTLD management and may worsen the prognosis.

Access to healthcare resources, including the availability of specialized diagnostic and therapeutic interventions, significantly impacts prognosis. Patients in resource-limited settings may face barriers to timely diagnosis, adequate treatment, and supportive care, resulting in poorer outcomes [19]. Socioeconomic factors, including poverty, inadequate housing, and a lack of social support, also influence prognosis by affecting disease management and access to healthcare services [4].

Impact on Quality of Life

PTLD exerts a profound impact on the quality of life of affected individuals, encompassing physical, social, and psychological dimensions [19]. Chronic respiratory symptoms such as cough, dyspnea, and recurrent infections impair physical function, restrict activities of daily living, and diminish exercise capacity, leading to functional limitations and disability.

Socially, PTLD may isolate individuals from their communities due to the stigma associated with TB, respiratory symptoms, and functional impairment. Social and occupational roles may be disrupted, leading to unemployment, financial strain, and reduced social participation. Psychological distress, including anxiety, depression, and post-traumatic stress disorder (PTSD), is common among PTLD patients, stemming from the chronicity of the disease, fear of recurrence, and perceived loss of control over one's health [19].

Emerging therapies

Emerging research on novel therapies holds promise for improving outcomes in PTLD. Targeted anti-fibrotic agents such as pirfenidone and nintedanib have shown efficacy in slowing disease progression and preserving lung function in patients with idiopathic pulmonary fibrosis (IPF), a condition characterized by similar fibrotic changes as PTLD [26]. These agents may hold potential for PTLD patients with progressive fibrosis.

Immunomodulatory therapies, including biologics targeting specific inflammatory pathways, are being investigated for their potential to modulate immune responses and attenuate tissue damage in PTLD. Cellular therapies, such as mesenchymal stem cell transplantation, hold promise for promoting tissue repair and regeneration in fibrotic lungs [27].

Nevertheless, the prognosis of PTLD is influenced by a complex interplay of factors spanning lung pathology, comorbidities, and healthcare access. PTLD exerts a significant toll on quality of life, impacting physical, social, and psychological well-being. Emerging research on novel therapies offers hope for improving outcomes and enhancing the quality of life in PTLD patients, highlighting the importance of continued research and innovation in this field.

Prevention and public health implications

Prevention of PTLD requires a comprehensive approach encompassing TB control programs, strategies for early detection and treatment of TB, and targeted public health interventions aimed at high-risk populations. Addressing these aspects is essential for reducing the burden of PTLD and improving long-term outcomes for TB survivors.

Importance of TB Control Programs

TB control programs play a crucial role in reducing the incidence and prevalence of PTLD by preventing active TB infection and optimizing TB treatment outcomes. Key components of TB control programs include early case detection through active case finding and passive case detection strategies, prompt initiation of standardized TB treatment regimens, and implementation of infection control measures to prevent TB transmission in healthcare and community settings. However, the literature suggests that in over 212 TB guidelines worldwide, only three international TB guidelines mention PTLD, with a paucity of ways to diagnose or treat it [5,28]. Moreover, the WHO End TB Strategy has not included PTLD, and the present TB registries record morbidity and mortality only during the course of treatment [5].

Enhanced case management and treatment adherence support are essential for ensuring successful TB treatment completion and reducing the risk of TB recurrence, which is a significant risk factor for PTLD development. Collaborative efforts between healthcare providers, public health authorities, and community stakeholders are necessary to strengthen TB control programs and mitigate the burden of PTLD. Migliori et al. put forth clinical standards to provide guidance on the assessment and management of PTLD and the implementation of pulmonary rehabilitation [19]. However, there is a paucity of data on PTLD worldwide [5].

Strategies for Early Detection and Treatment of TB

Early detection and treatment of TB are paramount for preventing PTLD development in TB survivors [28]. Public awareness campaigns promoting TB screening, symptom recognition, and healthcare-seeking behavior facilitate early case identification and the timely initiation of TB treatment [29]. Integration of TB screening services into primary healthcare settings, including clinics, hospitals, and community outreach programs, enhances access to TB diagnosis and treatment for at-risk populations [30].

Point-of-care diagnostic technologies, such as molecular assays and rapid TB tests, enable rapid and accurate diagnosis of TB, facilitating prompt initiation of appropriate treatment [31]. Implementing TB treatment monitoring and adherence support programs, including directly observed therapy (DOT) and patient education, promotes treatment completion and reduces the risk of TB recurrence and PTLD development.

Public Health Interventions Targeting High-Risk Populations

Public health interventions targeting high-risk populations, including TB survivors and individuals with comorbidities, are essential for preventing PTLD and mitigating its impact on public health. Post-TB follow-up care programs provide comprehensive medical assessment, monitoring, and management of TB survivors to detect and manage PTLD-related complications early [19].

Integration of PTLD surveillance systems into existing TB control programs enables monitoring of PTLD incidence, prevalence, and outcomes, facilitating evidence-based decision-making and resource allocation. Targeted interventions, such as smoking cessation programs, nutritional support, and pulmonary rehabilitation, address modifiable risk factors for PTLD and improve long-term health outcomes in TB survivors [19].

The prevention of PTLD hinges on effective TB control programs, early detection and treatment of TB, and targeted public health interventions targeting high-risk populations. By prioritizing TB prevention, early diagnosis, and comprehensive post-TB care, public health authorities can reduce the burden of PTLD and improve the health and well-being of TB survivors and at-risk populations.

Future directions

As PTLD continues to pose a significant clinical challenge, future research efforts should focus on advancing our understanding of its pathogenesis and optimizing management strategies. Additionally, exploring the potential role of personalized medicine approaches and fostering multidisciplinary collaboration are essential for improving outcomes in PTLD patients [5,19].

Areas of Research Needed

Elucidating the underlying pathogenic mechanisms driving PTLD development and progression is paramount for identifying novel therapeutic targets and interventions. Research efforts should focus on characterizing the immunological, inflammatory, and fibrotic processes underlying PTLD pathogenesis, including the role of host-pathogen interactions, immune dysregulation, and tissue remodeling [5,19,28].

Investigating the natural history of PTLD, including long-term outcomes and prognostic factors, is essential for understanding disease progression and identifying predictors of adverse outcomes. Longitudinal studies tracking PTLD patients over time can provide insights into the trajectory of lung function decline, disease exacerbations, and mortality rates, guiding clinical management and resource allocation [19,28].

Exploring the impact of environmental and genetic factors on PTLD susceptibility and progression may uncover novel biomarkers and therapeutic targets. Genetic studies investigating host susceptibility genes and gene-environment interactions may identify individuals at increased risk of developing PTLD and inform personalized treatment approaches.

Potential Role of Personalized Medicine Approaches

Personalized medicine approaches, including pharmacogenomics, biomarker-based risk stratification, and tailored treatment regimens, hold promise for optimizing PTLD management. Genetic profiling of PTLD patients may identify genetic variants associated with treatment response, adverse drug reactions, and disease progression, enabling personalized treatment selection and dose optimization [5].

Biomarker discovery efforts aimed at identifying novel blood, sputum, or imaging biomarkers predictive of disease activity, progression, and treatment response may facilitate early diagnosis, monitoring, and risk stratification in PTLD patients [32]. Integration of multi-omics data, including genomics, transcriptomics, proteomics, and metabolomics, may uncover molecular signatures underlying PTLD pathogenesis and inform precision medicine approaches [33].

Importance of Multidisciplinary Collaboration

Multidisciplinary collaboration among pulmonologists, infectious disease specialists, radiologists, and public health experts is essential for optimizing PTLD management and improving patient outcomes. Integrated care models that facilitate communication, coordination, and shared decision-making among healthcare providers enhance patient-centered care delivery and treatment adherence [19].

Collaborative research networks and consortia focusing on PTLD research and clinical trials provide platforms for knowledge exchange, data sharing, and research collaborations, accelerating scientific discovery and innovation in the field. Interdisciplinary research teams combining expertise in immunology, pulmonology, microbiology, radiology, and epidemiology are essential for addressing the complex challenges posed by PTLD and advancing patient care [19].

The future research directions in PTLD should prioritize understanding disease pathogenesis, exploring personalized medicine approaches, and fostering multidisciplinary collaboration to improve outcomes in PTLD patients. By addressing these areas, researchers and clinicians can advance their understanding of PTLD and develop effective strategies for its prevention, diagnosis, and management.

## Conclusions

PTLD is a significant consequence of TB treatment, posing global challenges. Diagnosis is complex, often resembling other lung diseases, requiring a comprehensive approach. Management involves tailored strategies, including medication, rehabilitation, and surgery. Gaps persist in understanding PTLD's origins and optimal treatment. Personalized medicine shows promise for tailored treatments. Multidisciplinary collaboration is crucial for advancing PTLD research and care. Strengthening TB control efforts and early detection are vital for PTLD prevention. Public health interventions are needed to mitigate PTLD's impact on individuals and communities. By addressing these challenges, the lives of TB survivors worldwide can be improved.

## References

[1] Bloom BR, Atun R, Cohen T (2017). Tuberculosis. Major Infectious Diseases, 3rd edition.

[2] (2024). WHO: Tuberculosis. https://www.who.int/health-topics/tuberculosis.

[3] Shanmuga KP, Parivakkam Mani A, Geethalakshmi S, Yadav S (2024). Advancements in artificial intelligence for the diagnosis of multidrug resistance and extensively drug-resistant tuberculosis: a comprehensive review. Cureus.

[4] Yadav S (2024). Understanding the complexities of post-tuberculosis lung disease: implications for global health. IP Indian J Immunol Respir Med.

[5] Singh S, Allwood BW, Chiyaka TL (2022). Immunologic and imaging signatures in post tuberculosis lung disease. Tuberculosis (Edinb).

[6] Allwood BW, Byrne A, Meghji J, Rachow A, van der Zalm MM, Schoch OD (2021). Post-tuberculosis lung disease: clinical review of an under-recognised global challenge. Respiration.

[7] Mpagama SG, Msaji KS, Kaswaga O (2021). The burden and determinants of post-TB lung disease. Int J Tuberc Lung Dis.

[8] Malefane L, Maarman G (2024). Post-tuberculosis lung disease and inflammatory role players: can we characterise the myriad inflammatory pathways involved to gain a better understanding?. Chem Biol Interact.

[9] Ravimohan S, Kornfeld H, Weissman D, Bisson GP (2018). Tuberculosis and lung damage: from epidemiology to pathophysiology. Eur Respir Rev.

[10] Stek C, Allwood B, Walker NF, Wilkinson RJ, Lynen L, Meintjes G (2018). The immune mechanisms of lung parenchymal damage in tuberculosis and the role of host-directed therapy. Front Microbiol.

[11] Wynn TA (2008). Cellular and molecular mechanisms of fibrosis. J Pathol.

[12] Bird K, Memon J (2023). Bronchiectasis. StatPearls [Internet].

[13] Hashem MK, Nasim YS, Mohamed-Hussein AA, Shaddad AM (2022). The clinical and functional characteristics of bronchiectasis among tuberculosis patients in Upper Egypt: a single-center study.

[14] Ciurkiewicz M, Herder V, Beineke A (2020). Beneficial and detrimental effects of regulatory t cells in neurotropic virus infections. Int J Mol Sci.

[15] Gai X, Cao W, Rao Y (2023). Risk factors and biomarkers for post-tuberculosis lung damage in a Chinese cohort of male smokers and non-smokers: protocol for a prospective observational study. BMJ Open.

[16] Seedat UF, Seedat F (2018). Post-primary pulmonary TB haemoptysis - when there is more than meets the eye. Respir Med Case Rep.

[17] Johnson DH, Via LE, Kim P, Laddy D, Lau CY, Weinstein EA, Jain S (2014). Nuclear imaging: a powerful novel approach for tuberculosis. Nucl Med Biol.

[18] Ivanova O, Hoffmann VS, Lange C, Hoelscher M, Rachow A (2023). Post-tuberculosis lung impairment: systematic review and meta-analysis of spirometry data from 14 621 people. Eur Respir Rev.

[19] Migliori GB, Marx FM, Ambrosino N (2021). Clinical standards for the assessment, management and rehabilitation of post-TB lung disease. Int J Tuberc Lung Dis.

[20] Nightingale R, Carlin F, Meghji J (2023). Post-TB health and wellbeing. Int J Tuberc Lung Dis.

[21] Nair GB, Ilowite JS (2012). Pharmacologic agents for mucus clearance in bronchiectasis. Clin Chest Med.

[22] Seo W, Kim HW, Kim JS, Min J (2024). Long term management of people with post-tuberculosis lung disease. Korean J Intern Med.

[23] Budweiser S, Moertl M, Jörres RA, Windisch W, Heinemann F, Pfeifer M (2006). Respiratory muscle training in restrictive thoracic disease: a randomized controlled trial. Arch Phys Med Rehabil.

[24] Halezeroğlu S, Okur E (2014). Thoracic surgery for haemoptysis in the context of tuberculosis: what is the best management approach?. J Thorac Dis.

[25] Swanson KL, Johnson CM, Prakash UB, McKusick MA, Andrews JC, Stanson AW (2002). Bronchial artery embolization : experience with 54 patients. Chest.

[26] Finnerty JP, Ponnuswamy A, Dutta P, Abdelaziz A, Kamil H (2021). Efficacy of antifibrotic drugs, nintedanib and pirfenidone, in treatment of progressive pulmonary fibrosis in both idiopathic pulmonary fibrosis (IPF) and non-IPF: a systematic review and meta-analysis. BMC Pulm Med.

[27] Song N, Scholtemeijer M, Shah K (2020). Mesenchymal stem cell immunomodulation: mechanisms and therapeutic potential. Trends Pharmacol Sci.

[28] van Kampen SC, Wanner A, Edwards M, Harries AD, Kirenga BJ, Chakaya J, Jones R (2018). International research and guidelines on post-tuberculosis chronic lung disorders: a systematic scoping review. BMJ Glob Health.

[29] Nyasulu P, Sikwese S, Chirwa T (2018). Knowledge, beliefs, and perceptions of tuberculosis among community members in Ntcheu district, Malawi. J Multidiscip Healthc.

[30] Zulu DW, Silumbwe A, Maritim P, Zulu JM (2022). Integration of systematic screening for tuberculosis in outpatient departments of urban primary healthcare facilities in Zambia: a case study of Kitwe district. BMC Health Serv Res.

[31] Hong JM, Lee H, Menon NV, Lim CT, Lee LP, Ong CW (2022). Point-of-care diagnostic tests for tuberculosis disease. Sci Transl Med.

[32] Wallis RS, Maeurer M, Mwaba P (2016). Tuberculosis--advances in development of new drugs, treatment regimens, host-directed therapies, and biomarkers. Lancet Infect Dis.

[33] Duffy FJ, Weiner J 3rd, Hansen S (2019). Immunometabolic signatures predict risk of progression to active tuberculosis and disease outcome. Front Immunol.

